# Variant in the 5′ Untranslated Region of Insulin-Like Growth Factor 1 Receptor Is Associated With Susceptibility to Mastitis in Cattle

**DOI:** 10.1534/g3.112.003095

**Published:** 2012-09-01

**Authors:** Mayumi Sugimoto, Yoshikazu Sugimoto

**Affiliations:** * National Livestock Breeding Center, Nishigo, Fukushima, 961-8511, and; † Shirakawa Institute of Animal Genetics, Nishigo, Fukushima, 961-8061, Japan

**Keywords:** IGF1R, autophagy, mastitis, FEZL

## Abstract

Mastitis is a common infectious disease of the mammary gland and generates large losses in the dairy industry. By means of positional cloning and functional analysis techniques, we here show that insulin-like growth factor 1 receptor (IGF1R) can possibly mediate susceptibility to mastitis through autophagy. Scanning the whole genome of cows (*Bos taurus*) that were susceptible or resistant to mastitis in the half-sib families revealed that susceptible cows had a relatively long stretch of cytosine residues (C stretch) in the 5′ untranslated region of *IGF1R*. The forebrain embryonic zinc finger-like (FEZL) transcription factor, which was previously identified as a factor controlling mastitis resistance in the same half-sib families, bound the C stretch of *IGF1R*. The susceptible type of FEZL with a glycine stretch containing 13 glycines (13G) and the longer C stretch of *IGF1R* together enhanced expression of IGF1R. Enhancing IGF1R inhibited autophagy in response to *Streptococcus agalactiae* invasion of mammary epithelial cells, whereas treatment with rapamycin, a known inducer of autophagy, rescued it. Cows carrying the variant combination of 13GFEZL might be more susceptible to mastitis as the result of impaired autophagy. Our results suggest that IGF1R could control innate immunity in mammals and serve as a potential tool for preventing mastitis.

Mastitis is an inflammation of the mammary gland caused by bacteria such as *Streptococcus agalactiae* and is a major problem in the dairy industry. Recently, we identified *forebrain embryonic zinc finger-like* (*FEZL*) as one of the genes responsible for susceptibility to mastitis (Sugimoto *et al.* 2006). The whole genome scan revealed a significant linkage to *Bos taurus* autosomal chromosome 21 (BTA21) and 22, the latter of which harbors *FEZL* (Sugimoto *et al.* 2006). The FEZL transcription factor contains C2H2-type zinc-finger domains and a glycine stretch within which susceptible cows have an insertion resulting in 13 glycines (13G) instead of 12 (12G). When cows are infected with mastitis, FEZL induces tumor necrosis factor-α and interleukin-8 through enhancing semaphorin 5A (Sugimoto *et al.* 2006). Because 12GFEZL promotes greater *semaphorin 5A* expression than 13GFEZL, the susceptibility to mastitis might result from differences in immune response.

Next we continued to search genes responsible for susceptibility to mastitis on BTA21 and found that insulin-like growth factor 1 receptor (IGF1R) would be considered a positional candidate gene. Insulin/insulin-like growth factor signaling (IIS) is an evolutionarily conserved process that controls the lifespan of organisms. Nematodes, flies, and mice with impaired IIS are relatively long-lived (Kenyon *et al.* 1993; Clancy *et al.* 2001; Holzenberger *et al.* 2003). IIS modulates resistance to challenges by heat shock, oxidative stress, ultraviolet damage, and pathogens (Murphy *et al.* 2003; Wolf *et al.* 2006). Long-lived *daf-2* mutants are resistant to bacterial pathogens, including *Pseudomonas aeruginosa*, *Enterococcus faecalis*, and *Staphylococcus aureus* (Garsin *et al.* 2003). Although some factors downstream of IIS, such as the antibacterial lysosyme lys-7 (Murphy *et al.* 2003), and the nuclear coregulator of daf-16, smk-1 (Wolf *et al.* 2006), have been shown to control innate immunity, it remains unclear how IIS modulates defense against invading bacteria.

Autophagy is another evolutionarily conserved process, through which the intracellular contents are digested to recover nutrients in response to starvation (Levine and Klionsky 2004). IIS controls autophagy in conjunction with changes in nutrient levels through phosphatidylinositol 3-kinase (PI3K) and target of rapamycin (TOR) signaling (Hay and Sonenberg 2004). An increase of class I PI3K products suppresses autophagy (Petiot *et al.* 2000). Inactivation of TOR, which is one of the downstream components of PI3K signaling, induces autophagy (Blommaart *et al.* 1995). Treatment with rapamycin, which is an inhibitor of TOR, also induces autophagy in yeast (Noda and Ohsumi 1998). It has been confirmed that class I PI3K signaling suppresses starvation-induced autophagy, whereas the loss of TOR activity induces autophagy even without starvation in flies (Scott *et al.* 2004). More directly, *daf-2* IGF1R mutants have been reported to show enhanced autophagy in nematodes (Mèlendez *et al.* 2003).

Surprisingly, in recent studies investigators have revealed that cells also deploy their autophagic machinery as a defense against invading bacteria (Levine 2005). When *Streptococcus pyogenes* entered the cytoplasm of nonphagocytic cells, the bacteria were selectively sequestered and killed by autophagy (Nakagawa *et al.* 2004). The induction of autophagy by starvation or treatment by rapamycin suppressed the intracellular survival of *Mycobacterium tuberculosis* (Gutierrez *et al.* 2004). In *C. elegans*, genetic knockdown of autophagy genes abrogates pathogen resistance (Jia *et al.* 2009). Thus, the activation of autophagy, leading to improved defenses against bacteria, might represent a role of IIS in innate immunity in mammals.

Here we demonstrate that cows that are susceptible to mastitis have 13GFEZL and a relatively long stretch of cytosine residues (C stretch) in the 5′ untranslated (5UTR) region of *IGF1R*. 13GFEZL and the longer C stretch of *IGF1R* together enhance expression of IGF1R in terms of both messenger RNA (mRNA) and protein levels. Enhancing IGF1R inhibited autophagy in response to *S. agalactiae* invasion of bovine mammary epithelial cells [BMECs (Rose *et al.* 2002)], suggesting that the susceptibility to mastitis might result from impaired autophagy. Our results thus demonstrate an unexpected role for the FEZL–IGF1R pathway in innate immunity in mammals.

## Materials and Methods

### Mapping

Initial mapping of quantitative trait loci (QTL) associated with mastitis resistance was performed as described previously (Sugimoto *et al.* 2006). We analyzed 181 susceptible and 297 resistant cows derived from sic half-sib Holstein sire families with a panel of 272 microsatellite markers covering all autosomes (BTA1–29) by using the average somatic cell score [SCS; log_2_ (somatic cell count /100) +3] during the first lactation period as a phenotype parameter because there is a strong genetic correlation between SCS and mastitis (Shook and Schutz 1994). For further mapping on BTA21, we genotyped with an additional 60 microsatellite markers, and newly developed 101 microsatellite markers as shown in supporting information, Table S1. Using the genotype and phenotype data as shown in Table S2, we performed QTL mapping based on the web-based software package QTL Express (Seaton *et al.* 2002), which implements the multimarker linear regression method (Knott *et al.* 1996). For weighting of FEZL, we included genotype of FEZL as a covariate. For correction of IGF1R and FEZL, we included genotype of FEZL and IGF1R as a fixed effect. Significance thresholds for the F statistic were derived at the chromosome and genome-wise levels on single-trait basis by the permutation test. A total of 10,000 random permutations of the data were performed.

### Quantitative polymerase chain reaction (qPCR)

RNA from the bovine mammary gland or transfected BMECs was extracted using Trizol (Invitrogen, Carlsbad, CA). QPCR was conducted with an ABI 7900HT sequence detection system using the comparative Ct method and glyceraldehyde-3-phosphate dehydrogenase as internal controls (Applied Biosystems). BMECs were kindly provided by H. Aso (Tohoku University, Sendai, Japan), and were cultured in Dulbecco’s modified Eagle’s medium supplemented with 20% fetal bovine serum (cat. no. 26140-079, lot no. 1007768; Invitrogen), 10 μg/mL transferrin, and 5 mM Na acetate. The bovine FEZL expression construct was as described previously (Sugimoto *et al.* 2006). Transfection was performed with Lipofectamine 2000 (Invitrogen) for 48 hr according to the standard Invitrogen protocols. Each qPCR was performed with nine replicate samples that were extracted from three transfection experiments and subjected to statistical analysis using the Student’s *t*-test. The primers used for QPCR were as shown in Table S3.

### Luciferase assay

The luciferase assay was performed as described previously (Sugimoto *et al.* 2006). COS7 cells were obtained from the Riken Cell Bank (Tsukuba, Japan). Fragments of the IGF1R 5UTR region were constructed using PCR with the following primers: forward, 5′-GCAGTTTTTCCTCCCTCCTG-3′; and reverse, 5′-AAAACAGGAGTCCCCACAGCGAGGT-3′. They were then cloned into the pGL3 (R2.2)-basic vector (Promega, Madison, MI). COS7 cells were cotransfected with bovine FEZL expression construct.

### Gel mobility shift assay

The nuclear protein of COS7 cells transfected with FEZL was as extracted using the CelLytic NuClear extraction kit (Sigma-Aldrich, St. Louis, MO) and examined by the gel shift assay system (Promega). Protein concentration was measured by Bio-Rad protein assay using bovine serum albumin as the standard (Bio-Rad, Hercules, CA). Electrophoresis was done with 4–20% TBE gel (Invitrogen) for supershift assays and 6% retardation gel (Invitrogen) for competition assays. The probe and competitors used were as follows: 5′-CACCCCCCCCTTTTTTTTTTTTGA-3′ for 8C; 5′-CACCCCCCCCCTTTTTTTTTTTTGA-3′ for 9C; and 5′-CACCCCCCCCCCTTTTTTTTTTTTGA-3′ for 10C. Sigma-Aldrich produced a rabbit polyclonal antibody to FEZL by injecting FEZL peptide: CDKFAHPAPYAHKER.

### Genotyping of *FEZL* and *IGF1R*

The genotyping of *FEZL* was carried out by PCR and sequencing with the following primers: forward, 5′-TCCAAGACGCTGCTCAGTTA-3′; and reverse, 5′-CCACAGCCTGGTTGATGAC-3′. The genotyping of *IGF1R* was carried out by PCR and sequencing with the following primers: forward, 5′-GCAGTTTTTCCTCCCTCCTG-3′; and reverse, 5′-AAAACAGGAGTCCCCACAGCGAGGT-3′. Clinical mastitis was recorded as described previously (Sugimoto *et al.* 2011). The Veterinary Clinical Center, Tokachi NOSAI, recorded clinical mastitis in the Tokachi area of Hokkaido from 2003 to 2008. Cows diagnosed with mastitis and treated at least once a lactation period by veterinarians were considered affected. The Holstein Cattle Association of Japan, Hokkaido Branch, sorted the cows according to their sires and age. They collected 225,965 mastitis records from total 809,879 cows during these 6 years in this area. 50,599 mastitis records were from total 199,160 daughters of the 126 genotyped sires as shown in Table S4.

### Western blotting

Bovine mammary gland tissue was processed as described previously (Sugimoto *et al.* 2003). The total protein (20 μg) in the supernatants was separated using the Protein GeBaGel Electrophoresis System (Gene Bio-Application, Kfar-Hanagid, Israel), and was blotted using an ECL Plus Western Blotting Starter Kit (GE Healthcare, Buckinghamshire, UK). The blots were incubated with a rabbit polyclonal antibody to IGF1R (sc-713; Santa Cruz Biotechnology, Santa Cruz, CA) and the Can Get Signal Immunoreaction Enhancer Solution (Toyobo, Osaka, Japan). After detection, the blots were stripped by Restore Plus Western Blot Stripping Buffer (Pierce, Rockford, IL), and were incubated with a rabbit polyclonal antibody to actin (A2066; Sigma-Aldrich). For BMECs, the blots were incubated with a rabbit polyclonal antibody to LC3 (PD014 or PM036; Medical and Biological Laboratories, Nagoya, Japan) or a mouse monoclonal antibody to V5 (R960-25; Invitrogen) after treating as the manufacturer recommended.

### SNaPshot and quantitative analysis of allele ratios

SNaPshot was performed using SNaPshot multiplex kit (Applied Biosystems). The 5UTR region of *IGF1R* was amplified by PCR using amplification primers (forward, 5′- AGTGTTGTCGCCTTCGCCCT-3′; and reverse, 5′- CACTCGTGGGCCAGAGCGAGAGC-3′). Amplified PCR products were purified and analyzed using extension primer 5′- AGACTTTTTCTTTTCCTCCTCCACCACCCCCCCCC-3′. Subsequent extension with DNA polymerase added a single fluorescent triphosphate complementary to the nucleotide at the polymorphic site. The extended primers labeled with different fluorescent dyes were analyzed and the peak area ratios were calculated to measure the relative amount of DNA or complementary DNA (cDNA). For each mammary gland, peak area ratios were measured for both DNA and mRNA (cDNA). Assuming that the two alleles were present in equal amounts in genomic DNA, measured cDNA ratios were normalized to the average of genomic DNA ratios. For cDNA preparations, each mRNA was converted to cDNA in three separate experiments.

### Starvation protocol

To obtain starvation conditions, BMEC cells were washed three times with Hanks’ solution and incubated in the same solution for 1 hr at 37°. This kind of starvation *in vitro* has been known to induce autophagy as well as deprivation of food in mice for 24 hr (Mizushima *et al.* 2004).

### Infection protocol

*S. agalactiae* Lehmann and Neumann 1896 was obtained from the Riken Bioresource Center (Saitama, Japan). Bacteria that were grown until mid-log phase were harvested and washed twice with phosphate-buffered saline (PBS; pH 7.4). The number of bacteria was determined by measuring the number of colony-forming units (CFUs). At 3 days before infection, 2 × 10^5^ BMECs were seeded in BD BioCoat Fibronectin 24-well plates (BD, Franklin Lakes, NJ) or the Lab-tekII CC2 Chamber Slide System (Nunc, Roskilde, Denmark). The *S. agalactiae* bacteria were added to each well at a 1:50 multiplicity of infection. After 1 hr of incubation, the cells were washed with PBS and cultured in growth medium containing antibiotics (100 μg/mL gentamicin and 100 U/mL penicillin G) to kill extracellular bacteria for the indicated time period (postinfection).

### Fluorescence microscopy

The rat LC3 expression construct tagged with enhanced green fluorescent protein, LC3-pEGFP, was kindly provided by T. Yoshimori (Osaka University, Suita, Japan). Transfection in BMECs was performed with Lipofectamine 2000 (Invitrogen) for 48 hr according to the standard Invitrogen protocols. After infection, the chamber slides were rinsed in PBS and fixed in 4% paraformaldehyde for 15 min. The BMECs were rinsed in PBS and incubated with 1 mg/mL of Hoechst 33342 for 15 min. The cells were observed under Axioplan 2 and Axiovision 4 (Carl Zeiss, Oberkochen, Germany).

### CFU viability assay

The infected cells were washed twice with growth medium and lysed in sterile distilled water. Serial dilutions of the lysates were plated on Tryptic Soy Broth agar. The IGF1 treatment of cells was performed by incubation with 100–200 nM of IGF1 (JRH Biosciences, Inc., Lenexa, KS) for 24 hr before infection. The rapamycin treatment of cells was performed by incubation with 100 nM rapamycin (Sigma-Aldrich). The data represent the average number of CFUs ± SE of the recovered bacteria for three independent experiments.

## Results

**Figure 1:**
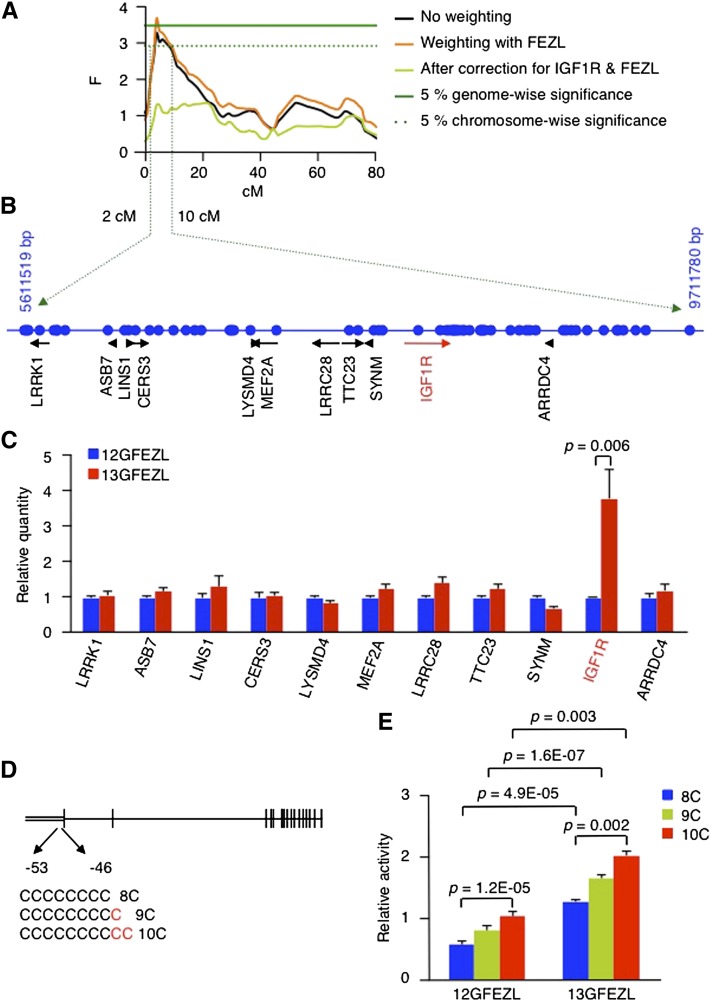
Susceptibility to mastitis is linked to IGF1R through FEZL. (A) F-statistic profile on BTA21 according to QTL express. (B) Schematic representation of genes (black) and microsatellite markers (blue dot) located in the candidate region. (C) Average expression level ± SE of genes located in the candidate region. 12GFEZL and 13GFEZL were transfected with the respective FEZL. *P* values were calculated by Student’s *t*-test. (D) Schematic representation of bovine *IGF1R* carrying polymorphisms on its 5UTR regions. The vertical and double lines indicate exons and the 5UTR region, respectively. (E) Average luciferase activity ± SE of *IGF1R* in COS7 cells transfected with FEZL. *P* values were calculated by Student’s *t*-test.

To identify the genes that influence susceptibility to mastitis on BTA21, we analyzed 478 cows from six half-sib Holstein sire families with an additional 161 markers and detected the QTL effect of SCS on 4 cM of BTA21 at the 5% chromosome-wise level (Figure 1A). Because FEZL helps modulate the innate immune response (Sugimoto *et al.* 2006), we computed the F-statistic for BTA21 by weighting with the genotype of FEZL to evaluate whether this transcription factor affected linkage in the chromosome. Surprisingly, the F-statistic for BTA21 by weighting with FEZL was increased, and the QTL was significant at the 5% genome-wise level (Figure 1A). This observation suggested a potential interrelationship between FEZL and the candidate genes on BTA21.

FEZL is a transcription factor and 12GFEZL and 13GFEZL have different effects on the expression of semaphorin 5A (Sugimoto *et al.* 2006). Therefore, the result shown in Figure 1A suggested that a gene influencing susceptibility to mastitis on BTA21 should fulfill the following criteria. First, the gene of interest should be located in the candidate region between 2 and 10 cM based on the 5% chromosome-wise significance. Second, the expression of the gene of interest should be controlled by FEZL. Third, 12GFEZL and 13GFEZL should have different effects on the expression of the gene of interest. Fourth, the gene of interest should carry a polymorphism that differs between alleles in resistant and susceptible animals. Fifth and finally, 13GFEZL should differentially affect the expression of the gene of interest depending on the polymorphic alleles. We then started to explore the candidate region in search of genes that fulfilled these criteria.

With respect to the first criterion, we identified 11 genes located in the candidate region based on the Bos_taurus_UMD_3.1/bosTau6 assembly (http://www.hgsc.bcm.tmc.edu/projects/bovine/; Figure 1B). To test whether these genes fulfilled the second and third criteria, we carried out qPCR of BMECs transfected with FEZL and verified that FEZL did not affect the expression of any of the 11 genes except for *IGF1R* (Figure 1C). As shown in Figure 1C, 13GFEZL enhanced the expression of *IGF1R* more than 12GFEZL, implying that *IGF1R* fulfilled the second and third criteria. In regard to the fourth criterion, we searched for polymorphisms of *IGF1R* that were carried by susceptible and resistant cows derived from our half-sib families and found a C stretch on the 5UTR region (Figure 1D). To test whether *IGF1R* fulfilled the fifth criterion, we performed luciferase assays using COS7 cells cotransfected with FEZL and luciferase vectors encoding the 5UTR region of *IGF1R* with different alleles. Consistent with the results of the QPCR of BMECs transfected with FEZL, 13GFEZL enhanced the luciferase activity of *IGF1R* with a long C stretch (Figure 1E). Based on the fulfillment of these five criteria, *IGF1R* appeared to be the most promising candidate gene, although we could not exclude the possibility that other genes on BTA21 might also influence resistance to mastitis. We also confirmed that the F-statistic reduced strongly after correction for IGF1R and FEZL interaction (Figure 1A).

**Figure 2:**
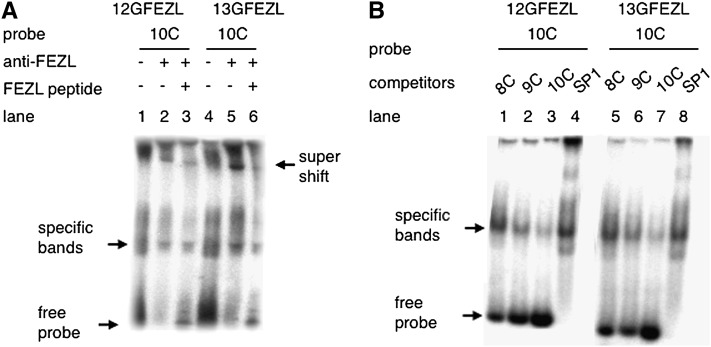
FEZL binds the C stretch in the 5UTR region of *IGF1R*, and enhanced IGF1R is linked to susceptibility to mastitis. (A) Gel mobility-shift assay of 12GFEZL (lanes 1–3) or 13GFEZL (lanes 4–6) with 10C. The specific binding indicated by the arrow was shifted by co-incubation with a rabbit polyclonal antibody to FEZL (lanes 2 and 5) Coincubation with FEZL peptide decreased super shifted complex (lanes 3 and 6). (B) Gel mobility-shift assay of 12GFEZL (lanes 1–4) or 13GFEZL (lanes 5–8) with 10C. The binding indicated by the arrow was reduced by coincubation with unlabeled 10C (lanes 3 and 7), but not by 8C (lanes 1 and 5) and SP1 (lanes 4 and 8).

The optimal binding sequence of FEZL was GCAG—not the C stretch—based on chromatin immunoprecipitation assays and sequencing (Sugimoto *et al.* 2006). Characterizations of transcription factor targets tend to be biased if DNA microarrays are not used (Iyer *et al.* 2001). Thus, FEZL might bind to the C stretch of *IGF1R*, and the different effects of the C stretch might have been due to their different binding abilities. To test this hypothesis, we performed a gel mobility-shift assay using COS7 cells transfected with FEZL. As expected, FEZL bound to the 5UTR region of *IGF1R*, and the specificity of binding was confirmed by gel supershift assays using the specific antibody (Figure 2A). We next assessed the binding ability of the C stretch of *IGF1R* more closely using competitors. Competition assays revealed that a long C stretch, 10C, bound stronger to FEZL (Figure 2B). Taken together, these findings indicate that FEZL binds to the 5UTR region of *IGF1R* and the length of the C stretch affects the binding ability.

**Figure 3:**
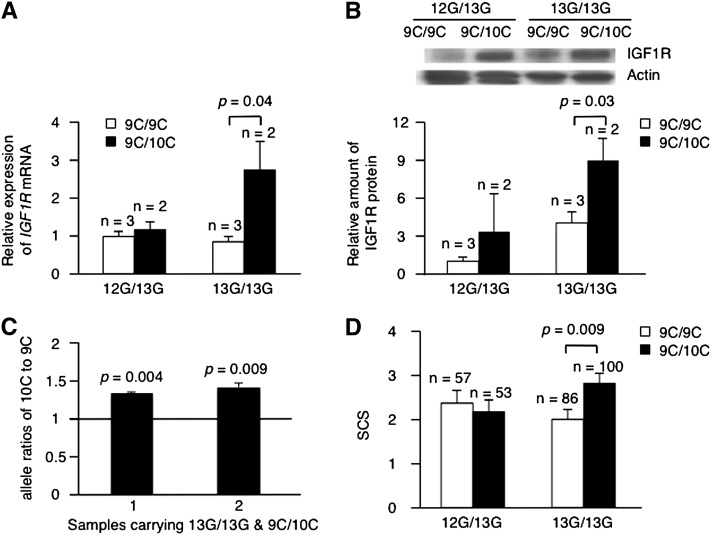
Enhanced IGF1R is linked to susceptibility to mastitis. (A) Average expression level ± SE in mRNA of IGF1R in bovine mammary gland. The ratios of IGF1R to glyceraldehyde-3-phosphate dehydrogenase relative to the sample of 12G/13GFEZL and 9C/9CIGF1R were shown. *P* values were calculated by Student’s *t*-test. (B) Representative Western blots and average amount ± SE in protein of IGF1R in bovine mammary gland. The ratios of IGF1R to actin relative to the sample of 12G/13GFEZL and 9C/9CIGF1R were shown. *P* values were calculated by Student’s *t*-test. (C) Average allele-specific expression level ± SE in IGF1R heterozygous bovine mammary gland. The ratios of 10C to 9C relative to genomic DNA of the sample were shown. *P* values comparing to the normal ratio (1) were calculated by Student’s *t*-test. (D) Average SCS ± SE values of cows derived from half-sib families. *P* values were calculated by Student’s *t*-test.

Cows carrying 13G/13GFEZL and *IGF1R* with a long C stretch showed greater expression of IGF1R at both the mRNA and protein level (Figure 3, A and B), demonstrating that the longer C stretch in combination with 13G/13GFEZL enhanced IGF1R *in vivo*. Even though there were only two samples carrying 13G/13GFEZL and 9C/10CIGF1R, which were collected randomly at a slaughter house, we also confirmed that the level of mRNA from the 10C allele yielded was greater than the 9C allele by determining the allelic mRNA ratio based on SNaPshot (Figure 3C). Enhanced IGF1R might affect resistance to infection in mammals, consistent with the findings of experiments on nematodes that *daf-2* mutants are resistant to bacterial pathogens (Garsin *et al.* 2003). Indeed, cows carrying 13G/13GFEZL and *IGF1R* with a longer C stretch had a greater SCS among 478 cows from six half-sib Holstein sire families (Figure 3D), indicating that they are susceptible to mastitis. Furthermore, we typed 126 commercially available Holstein sires in Japan (Table 1) and checked clinical mastitis records of cows derived from these sires. As expected, cows derived from sires carrying 13G/13GFEZL and *IGF1R* with a longer C stretch have a greater incidence of clinical mastitis (Table 2), suggesting that enhanced IGF1R is linked to slightly increased susceptibility to mastitis.

**Table 1:** The FEZL and IGF1R genotype of 126 commercially available Holstein sires in Japan

FEZL			
IGF1R	13G/13G	12G/13G	Total
7C/9C	1	0	1
8C/8C	2	0	2
8C/9C	3	5	8
8C/10C	5	0	5
8C/11C	0	3	3
9C/9C	50	0	50
9C/10C	22	6	28
9C/11C	19	1	20
10C/10C	3	0	3
10C/11C	6	0	6
Total	111	15	126

FEZL, forebrain embryonic zinc finger-like; IGF1R, insulin-like growth factor 1 receptor.

**Table 2:** The number of cows (% in parentheses) affected with mastitis in daughters of sires with 9C/9CIGF1R and 13G/13GFEZL compared with daughters of sires with 9C/10CIGF1R and 13G/13GFEZL grouped by age

Age, yr	<3 yr		Between 3 and 4 yr		Between 4 and 5 yr		Between 5 and 6 yr		More than 6 yr	
Sire	Affected	Unaffected	Affected	Unaffected	Affected	Unaffected	Affected	Unaffected	Affected	Unaffected
Genotype										
9C/9C	4939	20,914	3488	9698	1743	3688	674	1134	329	574
IGF1R	(19.1)	(80.9)	(26.5)	(73.5)	(32.1)	(67.9)	(37.3)	(62.7)	(36.4)	(63.6)
9C/10C	5368	21,144	6217	14,767	4704	8555	2188	3401	920	1361
IGF1R	(20.2)	(79.8)	(29.6)	(70.4)	(35.5)	(64.5)	(39.1)	(60.9)	(40.3)	(59.7)
*P* value*^a^*	0.001		2E^−10^		9E^−06^		0.16		0.04	

IGF1R, insulin-like growth factor 1 receptor; FEZL, forebrain embryonic zinc finger-like.

a Calculated by the Fisher’s exact test.

**Figure 4:**
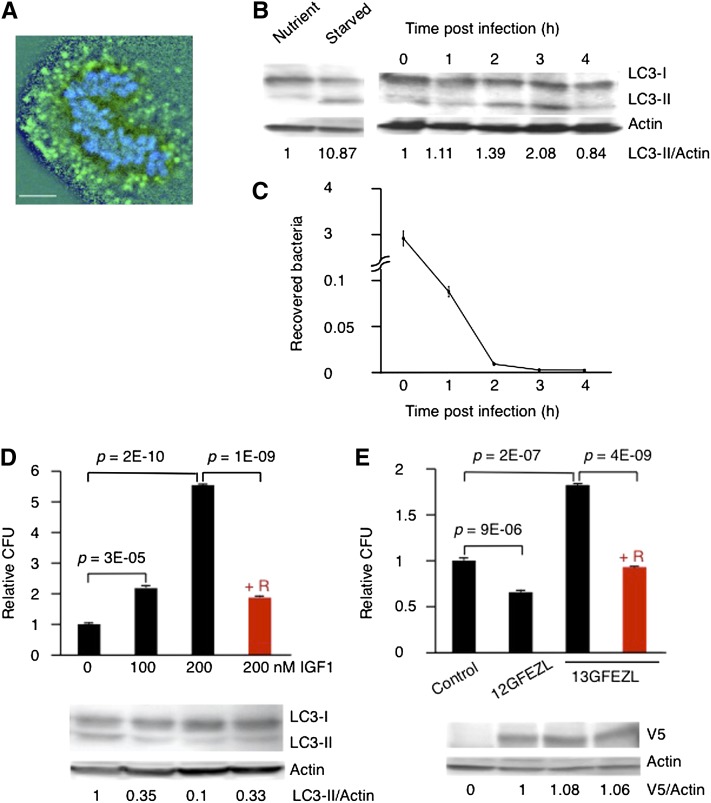
Enhanced IIS represses autophagy. (A) Confocal microscopic image of an autophagosome in BMECs at 1 hr after infection (LC3, green; DNA, blue). Bar, 10 µm. (B) Representative Western blots of LC3 in BMECs infected with *S. agalactiae*. The ratios of LC3-II to actin relative to nutrient BMECs or to BMECs 0 hr after infection were shown. (C) Viability of intracellular *S. agalactiae* in BMECs measured as the number of CFUs. The results represent the average ± SE of three independent experiments. (D) Viability of intracellular *S. agalactiae* in BMECs measured as the number of CFUs after treatment with IGF1 and representative Western blots of LC3. +R indicates treatment with rapamycin. *P* values were calculated by Student’s *t*-test. The ratios of LC3-II to actin relative to BMECs without IGF1 treatment were shown. (E) Viability of intracellular *S. agalactiae* in BMECs measured as the number of CFUs after transfection with FEZL and representative Western blots of V5. All samples were treated with 200 nM IGF1. +R samples were treated with rapamycin. *P* values were calculated by Student’s *t*-test. The ratios of V5 to actin relative to BMECs transfected with 12GFEZL were shown.

IIS controls starvation-induced autophagy through TOR and PI3K (Scott *et al.* 2004). An increase of class I PI3K products suppresses autophagy whereas inactivation of TOR induces autophagy (Blommaart *et al.* 1995; Petiot *et al.* 2000). Autophagy plays an important role in HeLa cells in fighting against invading *Streptococcus pyogenes* (Nakagawa *et al.* 2004). Thus, enhanced IIS might repress infection-induced autophagy in BMECs. To test this hypothesis, we initially investigated whether infection-induced autophagy was observed in BMECs. These are the first line of defense in protecting hosts from invading *S. agalactiae*, which is a major pathogen involved in mastitis. As expected, the infecting bacteria colocalized with LC3, which is an autophagosome-specific membrane marker (Nakagawa *et al.* 2004; Figure 4A). Figure 4B shows that starvation induced the amount of LC3-II (16 kD), which is a molecular form binding to autophagosome (Nakagawa *et al.* 2004). Infection also increased the amount of LC3-II in BMECs (Figure 4B). Moreover, the intracellular *S. agalactiae* had been killed in the BMECs 1 hr after infection (Figure 4C). Thus, the BMECs appear to defend the host against *S. agalactiae* through autophagy.

We next investigated the effects of IIS and FEZL on the autophagy of *S. agalactiae* in BMECs. As shown in Figure 4D, treatment with IGF1, which is a ligand of IGF1R, decreased the amount of LC3-II and increased the bacterial viability in the BMECs dose-dependently. On the other hand, treatment with rapamycin, which is an inhibitor of TOR and an inducer of autophagy, rescued this activity (Figure 4D). Moreover, transfection of 12GFEZL with a V5 tag repressed bacterial viability in BMECs (Figure 4E). By contrast, transfection of 13GFEZL increased the bacterial viability in BMECs, and treatment with rapamycin again rescued the autophagy (Figure 4E). There was no difference in the transfection efficiency between 12GFEZL and 13GFEZL (Figure 4E). These observations suggested that enhanced IIS repressed infection-induced autophagy in BMECs, and cows carrying 13G/13GFEZL and *IGF1R* with a longer C stretch might thus be slightly more susceptible to mastitis due to impaired autophagy.

## Discussion

There is accumulating evidence to suggest that lifetime exposure to infectious diseases influences human lifespan (Finch and Crimmins 2004; Christensen *et al.* 2006); however, the role of IGF1R in innate immunity in mammals has remained unclear, despite its influence on longevity in humans (Bonafè *et al.* 2003; Kojima *et al.* 2004; Suh *et al.* 2008) and mice (Holzenberger *et al.* 2003). Here we provide direct evidence that IGF1R is involved in innate immunity through autophagy in a mammalian species. In *Bos taurus*, a polymorphism in the 5UTR region of IGF1R was associated with mastitis incidence (Figure 1). This susceptible genotype of *IGF1R* is associated with its elevated expression and enhanced IGF1R inhibited autophagy in response to *S. agalactiae* invasion (Figure 4). Moreover, the polymorphisms in the autophagy genes are linked to genetic susceptibility to the inflammatory bowel disorder, Crohn’s disease, in human (Massey and Parkes 2007). Together, these data demonstrate a critical role for IGF1R through autophagy in mediating pathogen resistance in mammals.

One of the problems to identify genes by linkage analysis is that there would be many candidate genes in the critical region. Even if many single-nucleotide polymorphisms (SNPs) would have been developed in the critical region, the causative SNP using mapping families could not be identified because of its high linkage disequilibrium. Our results suggest that using interrelationships between a transcription factor and a gene of interest could be one of the solutions for this problem. Based on the fulfillment of our five criteria, we found that IGF1R could carry the causative SNP among 11 genes. Our five criteria might be arbitrary; however, we confirmed that correction for IGF1R and FEZL interaction reduced strongly the F-statistic (Figure 1A) and the effect of IGF1R on mastitis incidence was significant in independent samples (Table 2). Therefore, IGF1R could be the most promising candidate gene on BTA21.

We do not know how the SNP in the 5UTR region of IGF1R works *in vivo*. However, our gel mobility-shift assay and competition assays revealed that FEZL binds to the 5UTR region of *IGF1R* and the length of the C stretch affects the binding ability (Figure 2). The genotype of FEZL and IGF1R influences the expression level of IGF1R (Figure 3, A and B). *cis*-Acting polymorphisms would affect transcription, mRNA processing, mRNA stability, and protein translation (Wittkopp *et al.* 2004). Rapamycin has proven to suppress the translation of mRNAs that contain an oligopyrimidine tract at their transcriptional start (Jefferies *et al.* 1997). FEZL might modify the expression level of IGF1R through binding to its 5UTR region together with TOR signaling.

Although we confirmed that autophagy protected bovine mammary gland cells from invading *S. agalactiae*, whether autophagy protects mammary gland from other pathogens involved in mastitis such as *Staphylococcus aureus* and *Escherichia coli* should be clarified. It has been known that *S. pyogenes* and *M. tuberculosis* were killed by autophagy whereas *Shigella flexneri* was able to escape from autophagy ( Gutierrez *et al.* 2004; Nakagawa *et al.* 2004; Ogawa *et al.* 2005). Some reported that autophagic degradation was induced against *S. aureus* (Amano *et al.* 2006). On the other hand, others demonstrated that *S. aureus* escaped from autophagy (Schnaith *et al.* 2007). The role of autophagy for pathogens causative of mastitis in mammary gland other than *S. agalactiae* is yet to be resolved.

It is notable that autophagy is a mechanism for the elimination of damaged cells (Levine 2005). Mammary epithelial cells are involved in the phagocytosis of apoptotic cells during involution and in the regulation of inflammation (Monks *et al.* 2005). Impaired phagocytosis in mammary epithelial cells causes mastitis in mice (Hanayama and Nagata 2005). Autophagy might thus be an important mechanism in innate immunity, as a line of defense to protect hosts from invading bacteria.

As shown in Table 1, sires carrying susceptible variations of FEZL and IGF1R are common in Japan. By identifying one of the mechanisms underlying IGF1R-mediated immune function, we have discovered a potential new method for selecting cows in order to improve their health and welfare and also bring a great impact in a commercial setting.

## Supplementary Material

Supporting Information
