# Supplementary material for: Variant in the 5′ Untranslated Region of Insulin-Like Growth Factor 1 Receptor Is Associated With Susceptibility to Mastitis in Cattle
Source: G3 (Bethesda). 2012 Sep 1;2(9):1077–84. doi: 10.1534/g3.112.003095 (PMC3429922; doi:10.1534/g3.112.003095)
Supplement: Supporting Information [file supp_2_9_1077__index.html]

Supporting Information 

# Variant in the 5′ Untranslated Region of Insulin-Like Growth Factor 1 Receptor Is Associated With Susceptibility to Mastitis in Cattle

## Supporting Information for M. Sugimoto and Y. Sugimoto, 2012

**Files in this Data Supplement:**

- Table S1 - Information for newly developed microsatellite markers in BTA21 (.xls, 51 KB)
- Table S2 - Genotype and phenotype of 181 susceptible and 297 resistant cows (.xls, 818 KB)
- Table S3 - Primers used for QPCR (.xls, 37 KB)
- Table S4 - The number of cows affected with mastitis in daughters of 126 genotyped sires grouped by age (.xls, 57 KB)
